# Electropolymerisation Technologies for Next-Generation Lithium–Sulphur Batteries

**DOI:** 10.3390/polym15153231

**Published:** 2023-07-29

**Authors:** Soochan Kim, Youngkwan Lee

**Affiliations:** 1 Department of Engineering, University of Cambridge, Cambridge CB3 0FS, UK; 2 School of Chemical Engineering, Sungkyunkwan University, Suwon 16419, Republic of Korea

**Keywords:** electropolymerisation, conductive polymer, battery components, lithium–sulphur batteries

## Abstract

Lithium–sulphur batteries (LiSBs) have garnered significant attention as the next-generation energy storage device because of their high theoretical energy density, low cost, and environmental friendliness. However, the undesirable “shuttle effect” by lithium polysulphides (LPSs) severely inhibits their practical application. To alleviate the shuttle effect, conductive polymers have been used to fabricate LiSBs owing to their improved electrically conducting pathways, flexible mechanical properties, and high affinity to LPSs, which allow the shuttle effect to be controlled. In this study, the applications of various conductive polymers prepared via the simple yet sophisticated electropolymerisation (EP) technology are systematically investigated based on the main components of LiSBs (cathodes, anodes, separators, and electrolytes). Finally, the potential application of EP technology in next-generation batteries is comprehensively discussed.

## 1. Introduction

**Figure 1:**
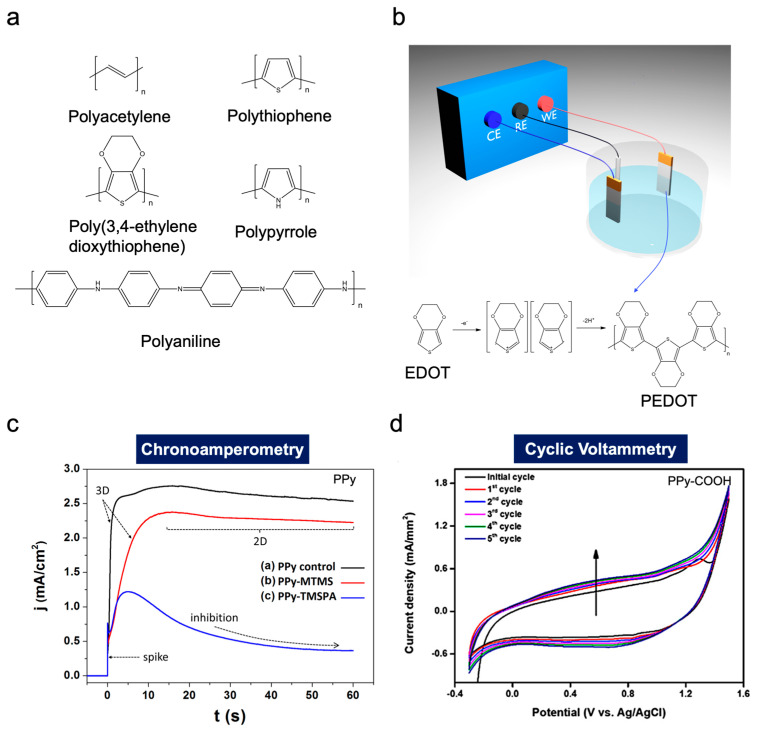
(**a**) Representative conductive polymers. (**b**) Schematic illustration of EP. (**c**,**d**) Electrochemical methods for EP; chronoamperometry [10] and cyclic voltammetry [11]. Reprinted with permission from [10,11]. Copyright 2023 MDPI and American Chemical Society.

Polymers are ubiquitous and associated closely to human life, such as in food, housing, transportation, and electric applications. In general, polymers are large molecules composed of repeated organic building blocks, and they were regarded as electrically insulating materials prior to the introduction of conducting polymers [1]. Conductive polymers exhibit unique electrical and optical properties, which are similar to those of inorganic semiconductors [2]. Polyacetylene, as a representative electrically conductive polymer with the simplest linear conjugated structures (Figure 1a), was discovered by Shirakawa, Heeger, and MacDiarmid and has been widely investigated to understand its application in microelectronics such as organic semiconductors [3]. The discoverers of polyacetylene were awarded the Nobel Prize in Chemistry (2000) [4]. In addition to polyacetylene, various conductive polymers such as polyaniline (PANI), polypyrrole (PPy), polythiophene, poly(3,4-ethylene dioxythiophene) (PEDOT), poly(para-phenylene), poly(phenylenevinylene), and polyfuran have been introduced into the core of next-generation energy, biological, and environmental applications [5,6].

Various approaches have been introduced to synthesise conductive polymers, such as chemical, electrochemical, photochemical, metathesis, concentrated emulsion, inclusion, solid-state, plasma polymerisation, and pyrolysis [7]. Among them, electropolymerisation (EP) is a cost-effective, facile, and rapid technique that can provide unique and sophisticated morphological structures depending on the applied polymerisation conditions (current density, potential, time, temperature, additives, etc.) [8]. In general, EP is initiated by the oxidation of a monomer in an electrochemical cell, and the initiated monomer is grown to the form of a polymer film on the surface of the operating electrode, such as an electric conductive metal, a conducting glass, or a carbonaceous substrate (Figure 1b) [8]. As a typical method, EP is performed by applying a potential difference or galvanostatic process (Figure 1c). These techniques are typically used for quantitative regulation by observing the nucleation mechanisms and growth. Other methods include potential dynamic techniques, such as cyclic voltammetry and linear sweep voltammetry (Figure 1d) [9]. These techniques involve a repetitive triangular potential waveform applied to the electrode, which is primarily used to obtain qualitative information regarding EP while the electrochemical behaviour of polymer films is investigated [9].

**Figure 2:**
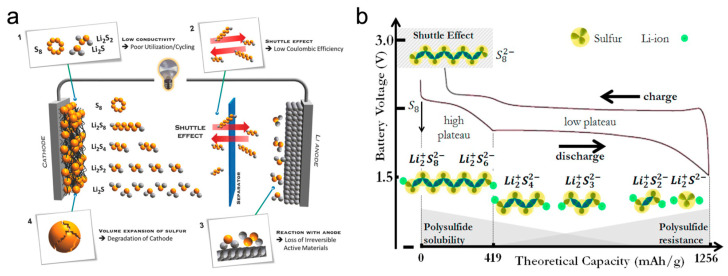
(**a**) Schematic illustration of main issues encountered in LiSBs [16] and (**b**) galvanostatic charging–discharging process of LiSBs [17]. Reprinted with permission from [16,17]. Copyright 2023 John Wiley and Sons, Inc. and Elsevier B.V.

EP is considered a promising technique for the fabrication of high-performance lithium–sulphur batteries (LiSBs) owing to their facile and controllable processes. LiSBs have been recognised as promising high-energy-density rechargeable batteries since their introduction in the 1960s [12]. Sulphur, as a cathode material, offers various advantages such as its low cost, abundance in nature, and high theoretical energy density (~2600 Wh Kg^−1^) [13,14,15]. However, the practical applications of LiSBs are impeded by challenges such as the low electrical conductivity of sulphur (~5 × 10^−28^ S m^−1^), the significant volumetric change (~80%) in sulphur upon lithiation, and lithium polysulphide (LPS) shuttle effects caused by the dissolution of intermediate LPS species during cycling (Figure 2). When the solid S_8_ accepts Li^+^ ions from the liquid electrolyte by electrochemical reaction, S_8_ will convert into long-chain LPSs. Long-chain LPS can dissolve into liquid electrolyte and diffuse to the anode side, losing the active material and increasing the viscosity of the electrolyte. Diffused LPS will react with the Li metal anode and form a solid-state short-chain LPS (Li_2_S or Li_2_S_2_) layer on the surface of the anode as a passivation layer [13,14,15]. Moreover, LPS shuttles, as the main issues hindering the practical application, are accelerated under practical conditions of LSBs such as high loading content of sulphur, low electrolyte–sulphur ratio (E/S ratio, μL mg^−1^) and low capacity ratio between the anode and cathode (N/P ratio). To suppress LPS shuttles, various strategies have been proposed based on the modification of the materials used for the cathode, electrolyte, separator, and anode [16,17,18,19]. For example, sulphur host design is based on nanostructured organic/inorganics, interlayers, electrolyte additives, a Li-alloy anode, and so on [18,19]. In particular, methods for trapping or obstructing LPSs during battery cycling have been extensively investigated, [15,16,17,18,19] and EP technologies have been considered promising for effectively controlling LPSs in the cathode and separator in a simple and sophisticated manner.

Herein, we review the recent advances and trends in the design of the main components of advanced LiSBs using EP technology. Applying EP technology to the typical components of batteries not only provides new insights into the design of next-generation LiSBs but also facilitates investigations into next-generation secondary batteries based on conversion-type electrodes.

## 2. Discussion

**Figure 3:**
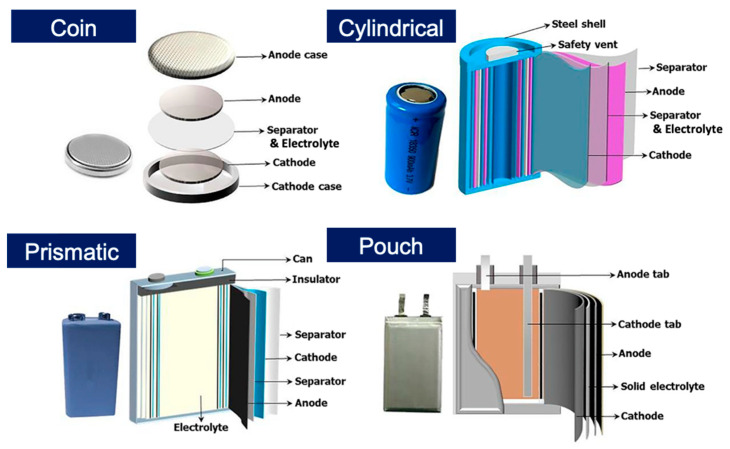
Schematic illustration of typical rechargeable battery configurations (coin, cylindrical, prismatic, and pouch type). Reprinted with permission from [23]. Copyright 2023 John Wiley and Sons, Inc.

Battery components typically include an anode, a cathode, an electrolyte, and a separator, all of which must function synergistically to realise practical applications (Figure 3). Various approaches for designing battery components have been introduced in LiSB research. Among them, cathode optimisation is the most effective method for addressing the main issues of LiSBs, i.e., LPS shuttles, low conductivity of active materials, and structural degradation of the cathode [20,21,22]. Various EP technologies have been introduced to facilitate cathode design.

### 2.1. Application in Cathode

In terms of the optimisation of sulphur cathodes, EP technologies have been reported as an effective strategy for designing cathode materials, sulphur hosts, and coating layers on the cathode. In the various conductive polymers, thiol-containing polymers are promising candidates for sulphur cathodes [24]. At temperatures above 180 °C, linear polysulfane can be obtained along the thiol surfaces through the ring-opening of cyclic S_8_ [14,20,24]. Thiol-based monomers cannot polymerise, but they can easily form copolymers with molten sulphur by connecting thiol groups and thiyl radicals. Consequently, sulphur is homogeneously distributed within the polymer matrix [24].

**Figure 4:**
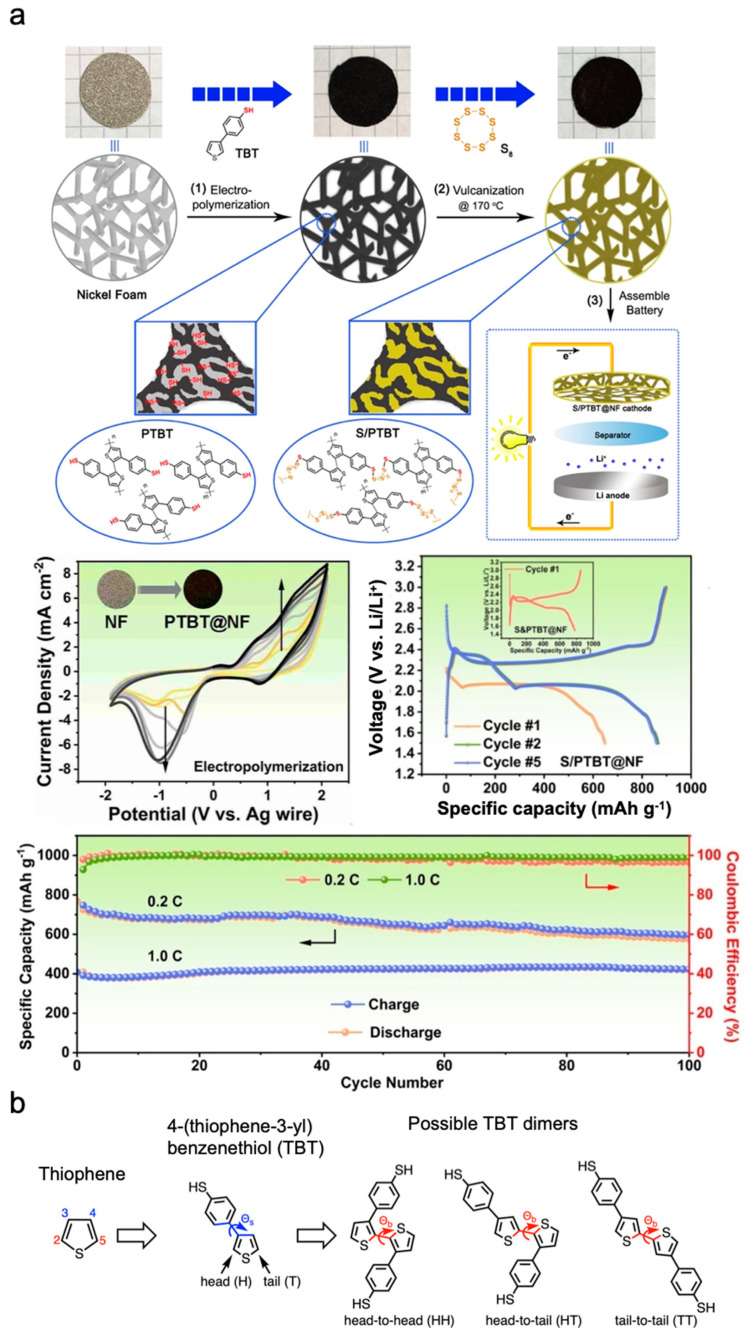
(**a**) Schematic illustration of fabrication process of PTBT and its electrochemical characterisations (cyclic voltammogram scan for polymerisation, galvanostatic charging–discharging curves of Li–S cell with PTBT, and battery cycling performance at 0.2 C and 1 C) [25]. (**b**) Proposed regiochemistry of electropolymerised PTBT [26]. Reprinted with permission from [25,26]. Copyright 2023 John Wiley and Sons, Inc. and American Chemical Society.

Ning et al. introduced a poly(4-(thiophene-3-yl) benzenethiol) (PTBT)-based organosulphur cathode, which was constructed via EP and vulcanisation without a binder or carbon additives (Figure 4a) [25]. A 4-(thiophene-3-yl)benzenethiol monomer was polymerised onto a nickel foam current collector via cyclic voltammetry to form porous and conducting PTBT frameworks, which served as chemical binding sites for LPSs and sites for sulphur hosts through vulcanisation. This strategy not only exploits the advantage of the chemical confinement of the conductive polymer for improving the performance of cathodes but it also simplifies the fabrication process of electrode materials for LiSBs. The S/PTBT cathode showed remarkable effects in suppressing LPS shuttles via covalent bonds between polymers and LPSs, and the assembled LiSBs showed stable long-term battery cycling performance over 200 cycles, with a low capacity fading rate of 0.178% per cycle (E/S ratio = 18.7; S-loading = 1.6 mg cm^−2^) [25]. 

Schütze et al. investigated the effect of the regiochemistry of an electropolymerised PTBT as an organosulphur cathode on its aggregation behaviour and charge transport (Figure 4b) [26]. The morphology of a polymer can significantly affect its conductive behaviour. Generally, polymers with more ordered and aligned structures provide more efficient charge transport, thus yielding higher conductivities compared with polymers with disordered structures [26,27]. Schütze et al. discovered that polymer chains can form crystalline phases only for a regioregular head-to-tail/head-to-tail regularity, whereas for a head-to-head/tail-to-tail regularity, the system did not show any long-range order after annealing. Furthermore, electronic structure simulations were performed to observe the mechanism by which the structural disorder in the head-to-head/tail-to-tail phases resulted in an energetic disorder that can potentially limit charge transport. Based upon the simulation results and the structural understanding obtained, their study provided new insights into the importance of polymer regularity and morphology modifications in the design of high-mobility crystalline phases, which allows the electrochemical performance of cathode materials for LiSBs to be optimised. In addition, this model can be used for more realistic cathode structures and geometries, including those that consider the effects of cross-linking sulphur between polymer chains as well as the degrees of freedom of the solvent and electrolyte [26].

As a sulphur host that can be prepared via EP, polycarbazole (PCBZ) is considered a promising material, owing to its electrical conductivity of 10^−2^–10^−3^ S cm^−1^, low monomer cost, high chemical stability, and porous structure [27,28,29]. The carbazole monomer can undergo EP from either the 3– and 6– or 2– and 7– positions, rendering it extremely versatile [27,30]. In addition, electropolymerised PCBZ (a p-type conducting polymer) maintains positively charged sites on its backbone because of the oxidative EP, which facilitates LPS adsorption, thus resulting in highly stable LiSB cycling.

Ramezanitaghartapeh et al. investigated the potential use of electrochemically polymerised PCBZ as a sulphur host and additive in sulphur cathodes [27]. By performing extensive characterisations using various electrochemical (cyclic voltammetry and chronoamperometry) and physicochemical techniques, an optimised PCBZ with high conductivity and a specific surface area was achieved in a LiClO_4_-acetonitrile electrolyte under a constant applied potential of +1.15 V (vs. Ag/Ag^+^). Additionally, Li–S cells (E/S ratio = 90; S-loading = 0.9 mg cm^−2^) fabricated using the prepared PCBZ in the cathode showed a high initial discharge capacity (~1600 mAh g^−1^), which was similar to the theoretical capacity at 0.1 C, as well as improved capacity retention. In particular, the nitrogen sites of PCBZ resulted in a strong electrostatic interaction with the Li electron acceptor of the LPS molecules, thus resulting in a high utilisation of active materials and regulated LPS shuttles with physical trapping of LPSs onto large specific areas of PCBZ [27].

**Figure 5:**
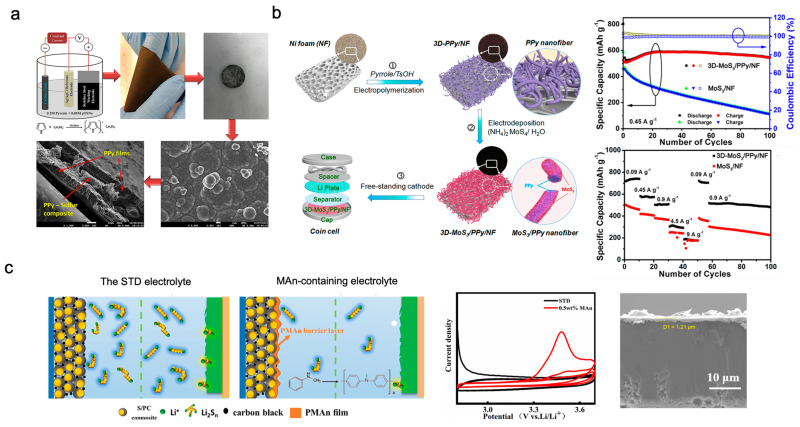
(**a**) Schematic illustration of fabricated freestanding PPy film and its SEM images (PPy film and cross-sectional image of sulphur cathode with PPy film) [31]. (**b**) Schematic illustration of fabrication process of 3D-MoS_3_/PPy electrode (MoS_3_ loading = 2 mg cm^−2^; 30 μL of the electrolyte amount) and battery cycling performances (rate capability and galvanostatic cycling at 0.45 A g^−1^) [33]. (**c**) Schematic illustration of electrochemical behaviours of MAn monomer, EP process, cross-sectional SEM image of sulphur cathode, and rate performance of Li–S cells [34]. Reprinted with permission from [31,33,34]. Copyright 2023 Elsevier B.V. and John Wiley and Sons, Inc.

The approach of coating the cathode surface and active materials via EP has been utilised in many studies on other conversion-type electrodes (metal chalcogenides, Si, metal fluorides, etc.) as well as sulphur cathodes. This approach allows the electrode surface to be controlled precisely based on the polymerisation conditions. Kaiser et al. fabricated a flexible freestanding PPy film via EP, which was then employed in a self-supported slurry-free sulphur-containing flexible electrode (Figure 5a) [31]. In the EP process, a freestanding PPy film was prepared by applying different current densities at a fixed charge density of 14.4 C cm^−2^ onto a stainless-steel plate (the operating electrode). Subsequently, the freestanding PPy film was peeled off from the stainless-steel plate and then attached to a powder-pressed sulphur cathode through mechanical pressing, which resulted in a sandwich structure. This sandwich electrode not only facilitated the activation of sulphur materials but also served as a barrier to minimise LPS shuttling and provided design flexibility and high areal capacity. In addition, the PPy film was a protective film that maintained the structural stability after many rounds of cycling and addressed the structural instability of the cathode caused by the significant volume expansion of sulphur. Thus, the fabricated sandwich electrode exhibited an initial discharge capacity of 804 mAh g^−1^ and stable cycling behaviour after 400 cycles, with a Coulombic efficiency of 93% (E/S ratio = 33; S-loading = 3 mg cm^−2^) [31]. Nakamura et al. coated PPy on a loaded three-dimensional (3D) current collector [32]. The PPy coating layer was prepared via oxidative EP with constant potential control in a three-electrode cell (a sulphur cathode, Pt wire, and Li foil as the working, counter, and reference electrodes, respectively) in the lithium bis(trifluoromethanesulfonyl)imide (LiTFSI)/glyme electrolyte. PPy was coated onto the cathode at a constant anodic potential of 4.5 V. The amount of charge transferred was 1.0 C cm^−2^, and the bath temperature was 80 C. The PPy layer on the 3D cathode exhibited efficient Li-ion selective permeability and outstanding suppression of LPSs, thus affording stable battery cycling even at high-rate operations (rate > 1 C). Thus, the developed Li–S cell presented a high discharge capacity of 794 mAh g^−1^ at 3 C (E/S ratio = 19; S-loading = 5 mg cm^−2^). This electrode architecture demonstrates the potential for imparting LiSBs with high-rate capabilities [32].

Yu et al. fabricated a freestanding cathode composed of MoS_3_/PPy nanowires deposited on a porous current collector using a two-step electrochemical method (Figure 5b) [33]. First, by controlling the concentration ratio of the Py monomer and soft-template molecules (0.1 M *p*-toluenesulfonic acid), PPy nanowires with a 3D network structure were successfully synthesised on the current collector by applying a current density of 2 mA cm^−2^ for 2000 s. Subsequently, MoS_3_ nanoparticles were decorated on the PPy through anodic electrodeposition, and the loading mass of MoS_3_ was adjusted based on the electrodeposition conditions. In this system, the formation of LPS was hindered by the driving cathode reaction mechanism of MoS_3_; however, the huge volume expansion of MoS_3_ during lithiation degraded the battery performance rapidly. The introduction of flexible conductive polymers into MoS_3_-based electrodes alleviated the structural degradation of the cathode due to the volume expansion of MoS_3_ and resulted in superior cycling performance compared with the performance of bulk-type MoS_3_ [33].

Shi et al. and Gao et al. developed in situ EP technologies for coating cathodes by adding monomers to the LiSB electrolyte, which differed from ex situ EP technologies for cathodes [34,35]. Additionally, Shi et al. proposed the use of N-methylaniline as an electrolyte additive in LiSBs (Figure 5c) [34]. A poly(N-methylaniline) (PMAn) surface coating was formed on the cathode surface via EP under approximately 3.55 V (vs. Li/Li^+^) during the initial battery cycling. After three cycles, the monomer in the electrolyte was completely polymerised and formed a protecting polymeric layer that measured approximately 1 µm thick on the sulphur cathode. The presence of PMAn effectively reduced the dissolution of LPS and alleviated its oxidation. Moreover, the prepared LiSBs (E/S ratio = 20; S-loading = 1–1.5 mg cm^−2^) exhibited significant improvements in terms of the battery cycling stability and battery capacity. Moreover, a battery with a 0.5 wt. % monomer additive delivered an initial specific capacity of 1224 mAh g^−1^ and after 200 cycles at 0.2 C, which was higher than that (59.8% capacity retention) achieved without the monomer additive [34]. Meanwhile, Gao et al. reported the in situ EP of EDOT for synthesising conductive PEDOT as a binder and coating layer for the cathode of a LiSB [33]. The EP of an EDOT-containing electrolyte at a potential of 4.10 V (vs. Li/Li^+^) for 800 s via chronoamperometry resulted in PEDOT:poly(4-styrenesulfonic acid) (PSS) or PEDOT:Li–PSS in the cathode containing PSS or in the Li–PSS pre-binder. LiSBs fabricated using this polymer (S-loading = 0.9–1.1 mg cm^−2^) showed significantly improved specific capacity, cycling stability, and rate performance compared with LiSBs without a conductive polymer and with an analogous commercial PEDOT:PSS binder in the cathode. The improvements in battery performance were attributed to the well-integrated interfaces between EDOT and other cathode materials, such as carbon conductive agents and sulphur active materials, where the LPSs trapped by PEDOT were arranged in close proximity to the carbon conductive agent to achieve efficient charge transfer [35].

### 2.2. Application in Anode

Li is well known as one of the most promising materials for anodes, owing to its ultrahigh capacity (3860 mAh g^−1^) and extremely low standard negative electrochemical potential [34,35]. However, LPS shuttles can corrode the surface of Li via the formation of a Li_2_S layer on the Li surface, thus resulting in low Coulombic efficiency and Li−S batteries with short cycle lives. In addition, the formation of an unstable solid–electrolyte interphase (SEI) by organic electrolytes and LPS shuttles can result in irregular Li plating/stripping and Li dendrite growth during repeated cycling [36,37,38,39]. Therefore, the formation of a stable SEI layer on the surface of Li that is corrosion-resistant is necessitated for realising high-performance LiSBs.

As a representative strategy, a protective layer is formed on the surface of Li, which should exhibit the following characteristics: chemical/electrochemical stability to electrolytes, high Li-ion diffusion rate, stability to LPSs, and flexible mechanical properties [38,40,41,42,43]. However, even if an ideal protective layer exists, the uniform coating on the surface of Li for constructing a stable interface between the protective layer and Li remains an issue to be addressed. In this regard, EP technologies are considered a promising strategy that can achieve uniform coverage on the surface of Li, depending on the EP conditions.

**Figure 6:**
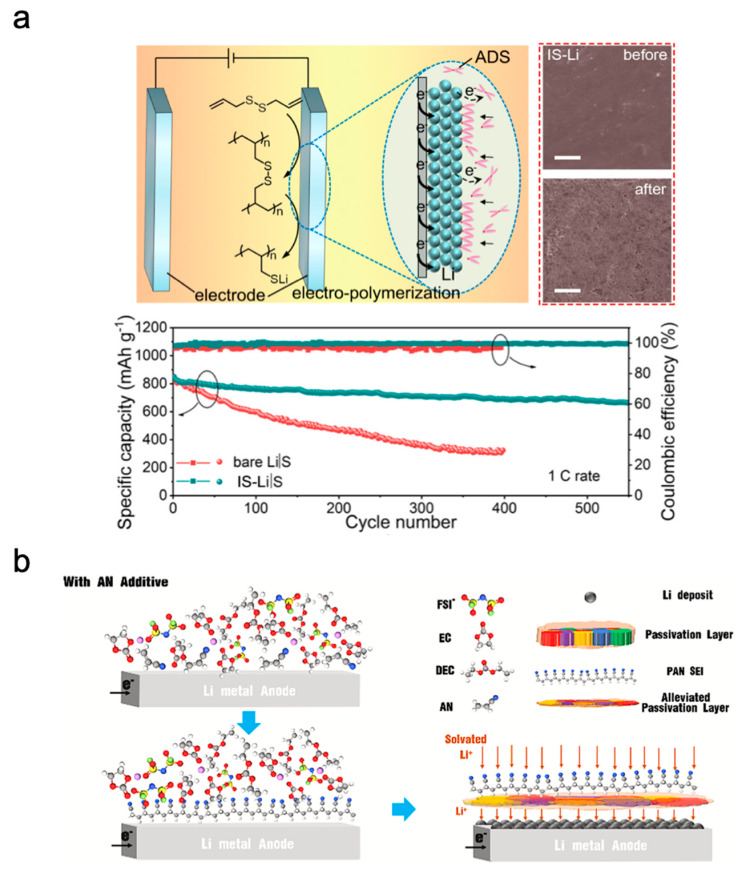
(**a**) Schematic illustration of rigid–elastic interfacial layers (poly allyl disulphide) on Li, SEM surface images after Li-symmetric cycling, and battery cycling performance at 1 C [38]. (**b**) Schematic illustration of regulating Li-dendrite by electropolymerised polyacrylonitrile layers onto Li metal anode [45]. Reprinted with permission from [38,45]. Copyright 2023 American Chemical Society and Elsevier B.V.

Liu et al. demonstrated the formation of a protective polymeric layer on the surface of Li by electrochemically regulating the polymerisation of allyl disulphides (Figure 6a) [38]. An allyl disulphide monomer was mixed with an ether-based electrolyte (1,3-dioxolane; DOL and 1,2-dimethoxyethane; DME) and LiTFSI salt; subsequently, this electrolyte was injected into a Li symmetric cell, which was discharged at a current of 0.5 mA cm^−2^ for 3, 10, and 20 h. Lithium isopropyl sulphide macromolecules, which formed a protective layer during EP and indicated different mechanical properties, ionic conductivities, and electrochemical stabilities. The protective layer deposited for 10 h presented the best balance. The ideal protective layer not only afforded sufficient mechanical strength to resist dendrite growth but also weakened the diffusion of LPS anions toward the Li metal, thus inhibiting their reduction to insulated Li_2_S on Li. Therefore, the resulting Li symmetric cell with the protective layer presented a voltage plateau and a low overpotential (~28.2 mV) at 2.0 mA cm^−2^ for 700 cycles. At a high current density (8.0 mA cm^−2^), the symmetric cell maintained its stable long-term Li plating/stripping behaviour. When the anode was paired with a sulphur cathode, the LiSBs (E/S ratio = 10; S-loading = 2.5 mg cm^−2^) showed an ultralong cycle life and high-rate capacity, thus demonstrating their promising application in LiSBs. Moreover, although they have not been applied to LiSBs, the protective layers on the surface of Li formed via the EP of PANI and polyacrylonitrile (Figure 6b), as reported by Xiong et al. [44] and Zhang et al. [45], are promising candidates.

### 2.3. Application in Separator

**Figure 7:**
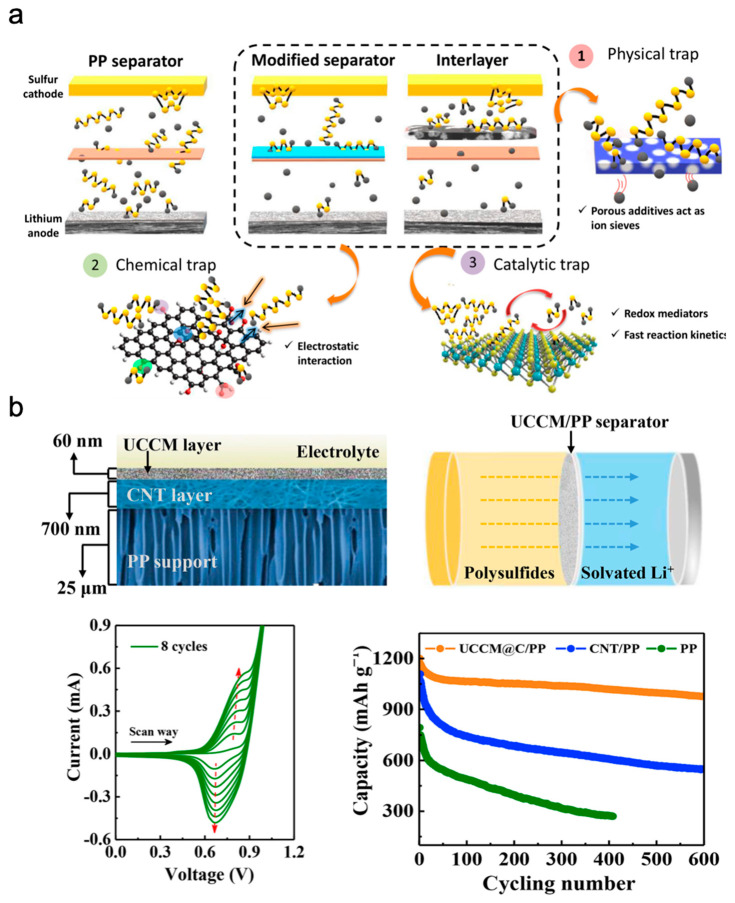
(**a**) Schematic illustration of modified separator and various polysulphides trapping mechanisms [51]. (**b**) Schematic illustrations of PCBZ-type interlayer, EP process of PCBZ via cyclic voltammetry, and battery cycling performance at 0.2 C [53]. Reprinted with permission from [51,53]. Copyright 2023 American Chemical Society and Elsevier B.V.

In conventional Li-ion batteries, the separators do not participate in electrochemical reactions; they provide physical separation between the cathode and anode and thus battery safety [37,46]. In the case of LiSBs, which exhibit severe issues, the separator must provide additional functions, such as suppression of the LPS shuttle via physical or electrochemical/chemical methods, improved mechanical properties, and enhanced electrolyte wettability [47,48]. Since the introduction of the concept of an interlayer between the electrodes and separator by Manthiram et al. in 2012, researchers have proposed inserting an interlayer and coating functional materials onto the separator, which has been shown to be a cost-effective and facile strategy for improving the cycling performance of LiSBs [49,50]. Inserting interlayers composed of organic or inorganic materials enhances the regulation of LPS shuttles and Li dendrite growth, thus resulting in stable long-term cycling performance (Figure 7a) [51]. However, loading/inserting organic or inorganic materials as an interlayer increases the thickness/weight, obstructs ion-transport pathways, and requires a significant amount of the electrolyte [37,52]. Therefore, the development of a thin functional interlayer with facile ion transport, similar to that of conventional separators, remains challenging.

EP technologies that can form thin and uniform interfaces on the separator based on EP conditions are regarded as a feasible approach for developing next-generation functional separators, and the electrical conductivity of CPs can expand the electrochemically active area for the conversion of diffused LPSs [54,55]. Guo et al. introduced a new EP strategy to develop an in situ PCBZ-type interlayer (Figure 7b) [53]. Using a carbon nanotube-coated commercial polypropylene separator and a TCB monomer in an electrolyte mixture (dichloromethane/acetonitrile, 3/2, *v*/*v*), they grew a PCBZ interlayer via cyclic voltammetry scanning at 50 mV s^−1^ between –0.8 and 1.03 V (vs. Ag/AgCl). The developed interlayer showed a porous morphology with uniform 0.82 nm nanochannels and an ultrathin thickness of 60 nm, which afforded facile ion transport and a high redox-active area as an interacting site for the LPSs. The LiSBs developed with this interlayer (E/S ratio = 10; S-loading = 2.1 mg cm^−2^) exhibited high sulphur utilisation and stable battery cycling, with a reversible capacity of 920 mAh g^−1^ after 600 cycles at 0.2 C. Even at high sulphur loading conditions, the Li–S cell showed remarkable battery cycling of 10 mAh cm^−2^ [53]. 

### 2.4. Application in Electrolyte

In LiSBs, ethers, including DOL and DME, are considered suitable solvents owing to their compatibility with LPSs [56,57,58]. Binary mixtures of electrolyte (1 M LiTFSI in DME/DOL, 1/1, *v*/*v*) are typically utilised to achieve LiSBs of low viscosity, high ionic conductivity, and favourable electrochemical performance [54,56]. However, LPS shuttles remain during battery cycling because of the high solubility of highly ordered Li_2_S_x_ (x ≥ 4), resulting from the Lewis basicity of the oxygen lone pair electrons of ethers and the Lewis acidity of the Li atoms of Li_2_S_x_ [56]. Therefore, a few methods have been proposed to control LPSs in electrolytes, such as the development of new Li salts and new mixtures of electrolyte solvents, the use of organic/inorganic additives, and the design of solid/quasi-solid-state electrolytes [57,59,60,61]. The use of new additives in electrolytes can form stable interfaces on the cathode and anode surfaces and inhibit LPS shuttles. Recently, the development of next-generation batteries to improve the overall battery safety and energy density through the use of solid/semi-solid electrolytes has received considerable attention [62,63,64]. For next-generation electrolytes, EP technologies have enabled the formation of stable interfaces between electrodes through the polymerisation of additives in the electrolytes and the development of polymer electrolytes.

**Figure 8:**
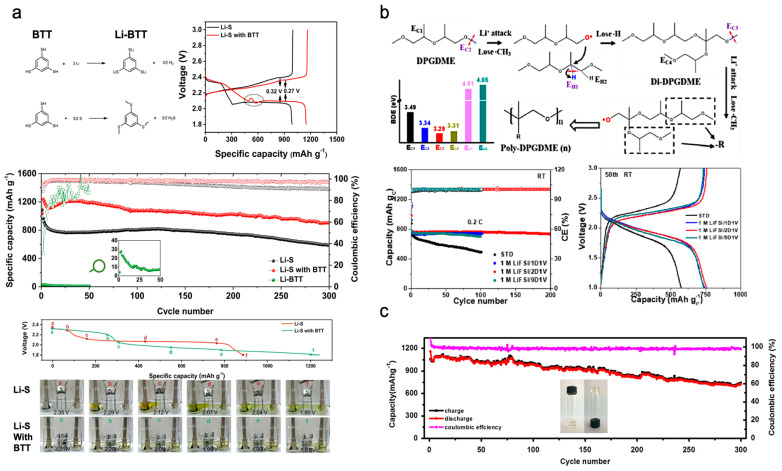
(**a**) Proposed reaction mechanisms of BTT, cyclic voltammograms scans of two types of Li–S cells at 0.05 mV s^−1^, battery cycling performance at 1 C, first discharge curves for Li–S visible cells, and photographs for the two types of cells at different discharge voltages [65]. (**b**) Polymerisation process of DPGDME and battery cycling performance, battery cycling performance at 0.2C, and galvanostatic charging–discharging profiles at 50th cycle [66]. (**c**) Battery cycling performance of Li–S cell with a novel quasi solid-state electrolyte (at 100 mA g^−1^); inset photograph shows the polymerised electrolyte [67]. Reprinted with permission from [65,66,67]. Copyright 2023 Springer Nature and Elsevier B.V.

Guo et al. presented a bifunctional electrolyte additive, i.e., 1,3,5-benzenetrithiol (BTT), which was used to construct solid–electrolyte interfaces on both electrodes via in situ organothiol transformation (Figure 8a) [65]. BTT not only reacted with Li to form lithium 1,3,5-benzenetrithiolate (Li-BTT), which was deposited on the anode, thus enabling reversible lithium deposition/stripping, but also formed an oligomer/polymer SEI on the cathode, which enabled the regulation of LPS shuttles. During battery cycling, the LiSBs with BTT exhibited three discharge voltage plateaus (a plateau at 2.4 V, a small peak and plateau at 2.3–2.1 V, and a long plateau at 2.1 V), unlike conventional LiSBs. At the additional plateau in the 2.3–2.1 V region, the LiSB with BTT underwent additional reactions (the S–S bonds of the BTT oligomer were formed during the recharge process break and were bound with Li-ions and electrons, which resulted in Li-BTT and Li_2_S) and the conventional conversion of Li_2_S_x_ (4 ≤ x ≤ 8) [20,65,66,67,68,69]. These processes caused the oligomerisation of BTT, altered the sulphur redox pathway, and prevented shuttle effects. The LiSBs protected by the BTT additive (S-loading = 0.88–2.65 mg cm^−2^) demonstrated high discharge capacity and stable cycling performance with high Coulombic efficiency [65].

Chen et al. demonstrated a nontoxic and nonflammable ether solvent, i.e., dipropylene glycol dimethyl ether (DPGDME), and its conversion to a polymer through in situ EP during cycling, as illustrated in Figure 8b [66]. In an electrochemical environment, DPGDME is attacked by Li-ions and produces a C–O bond rupture. The generated DPGDME• radical attacks methine, where the active-H descends to form di-DPGDME. [66] The dipolymer loses –CH_3_ concurrently, and poly DPGDME is formed. The formed poly-DPGDME effectively mitigates interfacial side reactions and adjusts the interphase with low polarisation, particularly on the sulphur cathode [66]. The assembled Li–S cells (S-loading = 2.5–11.6 mg cm^−2^) showed high sulphur utilisation exceeding 98% (1645.3 mAh g^−1^) and rapid charging (10 C) owing to the polyether-rich cathode SEI layer with rapid Li-ion diffusion. Moreover, the Li–S cells presented remarkable stable cycling, with 99.5% capacity retention over 400 cycles and an average Coulombic efficiency exceeding 99.9% [66].

Zhong et al. introduced a novel quasi-solid-state electrolyte comprising high-concentration salts and a polymer electrolyte for LiSBs (Figure 8c) [67]. In this system, the polymer electrolyte was prepared via the EP of DOL. EP was performed under potentiostatic control at an open-circuit voltage with a current of 0.1 mA, followed by a constant voltage step at 4.5 V (vs. Li/Li^+^) for 24 h. For application to LiSBs, the developed quasi-solid-state electrolytes not only inhibited the diffusion of LPS anions, but also protected the Li metal anode. The Li–S cell prepared using this electrolyte (S-loading = 1.5 mg cm^−2^) exhibited excellent cycle performance with high Coulombic efficiency (>98%) and cyclic stability, which afforded a capacity of 720 mAh g^−1^ after 300 cycles and did not require any additives. In addition, the Li–S cell maintained 99.8% of its initial capacity even after two weeks of storage, and self-discharge at room temperature was alleviated significantly [67]. Therefore, this quasi-solid-state electrolyte prepared via EP is a facile and promising candidate for achieving stable cycling behaviour as well as mitigating the self-discharge issue of LiSBs.

## 3. Conclusions and Perspectives

This article reviews the research progress on the EP technologies for LiSBs according to the battery components. EP can not only simplify the processes involved but also enable refined processes depending on the various EP conditions (method, potential, time, electrolyte, in situ process, etc.). The electropolymerised conductive polymer played various roles, including as an active material, a coating layer, a separator, an additive for a liquid electrolyte, and a precursor for polymer electrolytes, and when used in LiSBs as the coating layers onto the separator and additives in the electrolyte, developed LiSBs showed the best battery performance. However, for the practical application of EP technologies, it is necessary to conduct the following research. First, since the battery’s components are composed of various materials, it is required to investigate the mechanisms related to the surface adsorption of monomers and the growth of polymers to form uniform polymeric layers at various interfaces. Second, most of the reported studies showed conductive polymers fabricated by EP technologies at the laboratory scale. For practical applications, the productivity and efficiency of EP technology must be improved. Third, most reported LiSBs with the EP technology performed battery cycling at a high E/S ratio. The E/S ratio should be five or less to achieve practical LiSBs with high energy density [68,69,70], and to achieve this, polymers have to provide sufficient ion conductivity and improved control of the LPS shuttle.

As the next-generation platform in rechargeable batteries, all-solid-state batteries (ASSBs) have received significant attention because of their improved safety, high energy density, and LPS shuttle-free mechanism. ASSBs are composed of solid–solid interfaces in all components, unlike conventional Li-ion batteries with liquid electrolytes, which present various issues due to unstable interfaces, such as physical degradation due to unsatisfactory interfacial contacts and chemical/electrochemical decomposition [71,72,73]. In ASSBs, EP technology can be utilised to improve the physical contact between interfaces, form a protective layer on the electrode surface to suppress side reactions via chemical/electrochemical decomposition, and maintain structural stability. In addition, in a study regarding the replacement of Li metal anodes with Si anodes, electropolymerised polymers can prevent the extreme volume change in Si anodes and maintain electrical/ionic transport pathways based on their coating properties and flexibility, leading to stable cycling behaviours. Considering the versatility of EP, it can potentially become a key technology for addressing the issues of next-generation conversion-type battery materials, such as sulphur, chalcogenides, silicon, and fluorides [74,75,76,77].

## Data Availability

Not applicable.
